# Caregiver-Mediated Enhanced Milieu Teaching with Shared Book Reading Using Telepractice-Based Coaching

**DOI:** 10.3390/bs16081311

**Published:** 2026-08-02

**Authors:** Matthew Klein, Laci Watkins, Yusuf Akemoğlu, Anindita Banerjee, J. B. Ganz

**Affiliations:** 1 Department of Educational Psychology, Texas A&M University, College Station, TX 77840, USA; mjklein@tamu.edu (M.K.); anindita.banerjee@tamu.edu (A.B.); 2 Department of Special Education, Duzce University, Düzce 81620, Turkey; yusufakemoglu@gmail.com; 3 National AI Institute for Exceptional Children, University of Buffalo, Buffalo, NY 14260, USA; 4 Department of Special Education and Communication Disorders, University of Nebraska-Lincoln, Lincoln, NE 68503, USA; jganz3@unl.edu

**Keywords:** autism, caregiver implemented intervention, telehealth, EMT, book reading

## Abstract

Caregiver coaching is an established practice for training caregivers to implement interventions for young autistic children. Enhanced milieu teaching (EMT) and shared book reading are naturalistic interventions used to teach language to young autistic children. Telepractice as a training and coaching method provides an increased level of flexibility to caregivers and coaches in providing interventions. Using a non-concurrent multiple baseline design across caregivers, this study taught four caregiver–child dyads to implement an intervention package consisting of EMT with shared book reading. Caregivers were trained using a telepractice model using behavior skills training. Coaching was provided to the participating caregivers throughout the intervention until they reached mastery criteria. Due to attrition, only two caregivers reached final mastery criteria for intervention implementation. Target outcomes included caregiver fidelity of implementation and child unprompted target word use. Results indicated that training and coaching resulted in increases in caregivers’ implementation fidelity, with evidence of generalization. Mixed results for child target word use were observed, with a greater effect observed on target word use during reading than during play. Findings indicate that while caregivers can be taught to improve their use of naturalistic strategies, the synchronous coaching model may need modifications for feasibility.

## 1. Introduction

Autistic children may experience differences in social communication, including challenges initiating and maintaining reciprocal verbal exchanges, which are associated with later difficulties forming and sustaining social relationships (American Psychiatric Association, 2013; Prelock & Nelson, 2012). Autistic children may also demonstrate delays in higher-level forms of play such as symbolic or collaborative play (Holmes & Willoughby, 2005), and together these differences may limit opportunities for peer engagement and positive social development. As a result, many early interventions for autistic children explicitly target social communication or embed communication goals within broader intervention frameworks (Hume et al., 2021).

### 1.1. Naturalistic Developmental Behavioral Interventions

Naturalistic developmental behavioral interventions (NDBIs) are a commonly used class of early interventions for autistic children that draw from both applied behavior analysis and developmental science (Schreibman et al., 2015). NDBIs use core behavior-analytic principles, including prompting, environmental arrangement, and the antecedent–behavior–consequence framework, while emphasizing developmentally appropriate goal selection, child-led interactions, and instruction embedded within natural contexts. Their theoretical foundations include the work of Lovaas (1987), Vygotsky (1966/2016), and Piaget (1952), with Vygotsky’s emphasis on social interaction and scaffolding being particularly relevant to naturalistic intervention models.

### 1.2. Enhanced Milieu Teaching

Enhanced Milieu Teaching (EMT; Kaiser & Hester, 1994) is a naturalistic language intervention used to support communication development in young children with language delays and developmental disabilities. As an NDBI, EMT combines applied behavior analysis-based strategies, including environmental arrangement, balanced turn-taking, and a least-to-most prompting hierarchy (wait time, open-ended question, choice question, mand model), with a child-directed approach that increases child agency and motivation (Vivanti & Zhong, 2020). EMT has demonstrated effectiveness in increasing communication frequency and target vocabulary for children with language delays and autistic children (Hancock & Kaiser, 2002; Kaiser & Hester, 1994; Kaiser et al., 2000) and can be implemented by both clinicians and caregivers (Hemmeter & Kaiser, 1994; Kaiser et al., 2000). However, findings are not uniformly positive. Reviews of naturalistic developmental behavioral interventions indicate considerable variability in child outcomes, with intervention effects influenced by factors such as children’s developmental characteristics, intervention intensity, caregiver implementation fidelity, and opportunities for practice across natural environments (Schreibman et al., 2015). Similarly, although caregiver-implemented language interventions frequently improve caregiver strategy use, corresponding improvements in child communication are often smaller and less consistent across studies (Oono et al., 2013). These findings suggest that while EMT represents a well-supported naturalistic language intervention, additional research is needed to better understand the conditions under which caregiver-mediated EMT produces consistent child communication outcomes.

### 1.3. Shared Book Reading

Early language experiences are critical for later academic and language outcomes (Sénéchal & LeFevre, 2014), and early vocabulary skills are predictive of later language development (Marchman & Fernald, 2008). Shared book reading, defined as interactive exchanges between an adult and child in which the child is actively engaged during the reading experience (Noble et al., 2019), represents a particularly rich context for language input. The frequency of caregiver–child book reading is associated with gains in expressive and receptive vocabulary and stronger later academic outcomes (Barnes & Puccioni, 2017; Bracken & Fischel, 2008; Farrant & Zubrick, 2013; Roulstone et al., 2011; Sénéchal et al., 2008), and caregivers and children engage in higher rates of verbal interaction during shared book reading than in other daily routines (Gilkerson et al., 2017). Because shared book reading occurs frequently in the home, it provides an authentic context for embedding intervention strategies (Kaderavek, 2002).

Recent research suggests that NDBI strategies, including modeling target words, using prompting hierarchies, and following the child’s lead, can be effectively integrated into shared book reading (e.g., Akemoglu et al., 2020, 2022; McLeod et al., 2017; Towson et al., 2021). Although this integration is a relatively recent area of study, findings indicate it may be effective for increasing communication and target vocabulary acquisition for children with language delays, developmental disabilities, and autism (Akemoglu et al., 2022; Kim et al., 2020; McLeod et al., 2017). The combination of EMT and shared book reading is theoretically well-suited to producing vocabulary gains beyond what either approach might achieve alone. Shared book reading generates higher rates of verbal interaction than other daily routines (Gilkerson et al., 2017), provides a more structured, turn-taking context that may scaffold the caregiver–child exchanges EMT prompting hierarchies require (Clemens & Kegel, 2020), and repeated exposure to target words across readings reinforces EMT targets; caregivers also use more expansions, prompting, and questions during book reading than during play (Mathers et al., 2024).

### 1.4. Caregiver-Mediated Interventions

Early intervention services for young children are often delivered in the home, a context that can produce favorable outcomes in language and cognitive development for young children with disabilities, including autistic children (Meadan et al., 2016; Rickards et al., 2009). Caregiver-implemented interventions in this context can provide developmental benefits for children and improved self-efficacy in caregivers (Trivette et al., 2010), and caregivers of autistic children have been effectively taught and coached to deliver a variety of communication and behavioral interventions (Movahedazarhouligh, 2021).

Telepractice, the use of telecommunication technologies to support remote care (González-García et al., 2023), offers increased flexibility in service delivery and extends reach to families for whom in-person services may be impractical (Poole et al., 2022). For autistic children, caregiver-based interventions delivered via telepractice have been shown to be effective, resulting in communication gains for children, and caregivers report satisfaction and increased confidence with this delivery model (García-Grau et al., 2024; Garnett et al., 2019, 2022). Studies have also begun integrating naturalistic book reading into play routines that incorporate EMT strategies to teach target vocabulary to children with language delays and autistic children (Kim et al., 2020; McLeod et al., 2017), suggesting the efficacy of these approaches in combination.

However, no study to date has assessed an intervention package composed of naturalistic book reading with EMT during play when implemented by caregivers who were taught using telepractice models. Prior studies combining EMT with shared book reading used researcher- or clinician-implemented delivery (Kim et al., 2020; McLeod et al., 2017); prior caregiver-implemented EMT studies did not include shared book reading (Hemmeter & Kaiser, 1994; Kaiser et al., 2000); and while telepractice coaching for caregiver-implemented naturalistic interventions has shown promise (García-Grau et al., 2024; Garnett et al., 2019, 2022), none evaluated it for the EMT with shared book reading package. The present study is the first to combine caregiver delivery, the EMT with shared book reading package, and telepractice-based BST coaching, with unprompted target word use across reading and play as the child outcome.

Although caregiver-mediated interventions and telepractice have demonstrated considerable promise, findings across the literature have been more mixed than uniformly positive. A Cochrane review of parent-mediated interventions reported strong evidence for improvements in parent–child interaction but found inconsistent or inconclusive evidence for many distal child language and communication outcomes, highlighting the need for additional high-quality trials (Oono et al., 2013). More recent systematic reviews likewise suggest that caregiver-mediated interventions can improve social communication, but intervention effects vary according to child characteristics, intervention dosage, caregiver implementation fidelity, and study quality (Pacia et al., 2022). Similarly, telepractice has expanded access to caregiver coaching and is generally viewed as acceptable by families; however, researchers continue to identify challenges related to technology access, scheduling demands, caregiver workload, and maintaining participation throughout intervention, indicating that questions of feasibility remain alongside questions of efficacy (Pacione, 2022). These mixed findings suggest that, while telepractice-based caregiver coaching is promising, further research is needed to identify delivery models that balance intervention effectiveness with family feasibility and long-term participation.

The purpose of this study is to evaluate the efficacy of a caregiver-coaching telepractice model for teaching caregivers to implement naturalistic book reading procedures and EMT procedures for target vocabulary acquisition in young autistic children. The research questions are (a) can caregivers be taught to deliver an intervention package with high treatment fidelity, that consists of naturalistic book reading and EMT for their young autistic children? and (b) is there a functional relation between a caregiver-implemented intervention package consisting of naturalistic book reading and EMT procedures and child target vocabulary acquisition for young autistic children?

## 2. Method

### 2.1. Ethical Approval

Ethical approval was received from the first author’s university’s Institutional Review Board prior to beginning the study. All participating caregivers gave written consent for themselves and written permission for their children to participate. Caregivers were taught to gain assent from their children before each session and were instructed to discontinue a session if a child showed signs of withdrawing assent (e.g., verbal refusal, leaving the area).

### 2.2. Experimental Design

A non-concurrent multiple baseline design (NCMBL; Watson & Workman, 1981) across participants was used. This design provided recruitment flexibility and was appropriate because participants were not in shared settings (Slocum et al., 2022a, 2022b). The NCMBL was necessary because home-based telepractice required caregivers to record sessions independently, making concurrent data collection impractical, and because each dyad used individualized materials with no shared stimulus context. Threats to internal validity were minimized through three features: (a) participants had no contact with one another and were enrolled in separate home settings, (b) baseline lengths were varied across tiers (Slocum et al., 2022a, 2022b), and (c) tier assignment was determined randomly (Ledford & Gast, 2018). Study phases included baseline, caregiver instruction, implementation with coaching, independent implementation, and maintenance, with staggered entry into intervention based on random tier assignment.

### 2.3. Recruitment and Participants

Participants were recruited via social media and through disseminating recruitment materials to university listservs and at local clinics serving autistic children. Participants completed a form on Qualtrics to register interest and were contacted by a member of the research team to confirm eligibility. All participants came from one state in the southwestern United States. In order to participate, the caregiver needed to be at least 18 years of age, hold legal custody of the child in their dyad, reside in the United States, and be fluent in English. Child participants needed to be between the ages of three and eight years of age at the time of intake and be diagnosed with autism or suspected to be autistic and in the process of an autism evaluation. For three of the four children, a diagnosis of autism was reported by caregivers; the fourth child, Elias, was suspected to be autistic and was in the process of a formal evaluation at the time of enrollment, and no independently confirmed diagnosis was available for him. Four caregiver–child dyads met all inclusion criteria. All names are pseudonyms. The dyads were Theresa and Anne, Cheryl and Skyler, Mary and Elias, and Lucy and Gianna. All dyads spoke English in the home. Skyler was eight years of age at intake and turned nine during the course of baseline. Additional demographic information for participating dyads can be found in Table 1.

### 2.4. Setting

The intervention was delivered in the dyads’ homes in quiet, distraction-free spaces including living rooms, dining rooms, and bedrooms. All coaching sessions were conducted via Zoom, and caregivers recorded all baseline and intervention sessions using an internet-connected device through Zoom’s record feature. Participants were instructed to conduct the sessions in a distraction-free area, meaning an area free of extraneous noise (e.g., T.V., radio, video games, etc.), free of unrelated interactions (e.g., unrelated adults conversing, etc.), and free from distracting or unrelated materials (e.g., unrelated toys, etc.). The environment was assessed by the coder following each session to determine that it met these criteria.

### 2.5. Intake Procedures

The first author completed an intake interview with each family to assess reading habits, play routines, and child interests. Caregivers received guidance on recognizing child assent and withdrawing assent, completed the Social Communication Questionnaire (SCQ; Rutter, 2003) and the MacArthur Bates Communicative Development Inventory: Words and Sentences (CDI; Fenson, 2002). These assessments were given to provide a measure of autistic traits and current language level, respectively. These assessments were not used to confirm a diagnosis. Child assessment scores are reported in Table 2.

### 2.6. Researchers

The first author served as trainer and coach. He was a doctoral student in special education with a master’s degree in education, two years of classroom experience in a play-based early childhood special education program, and prior training in NDBIs.

### 2.7. Materials

Caregivers selected five themes from a provided list based on their child’s interests. Two books, one per theme, were selected with assistance from a children’s librarian, and three to five target words per book were confirmed as not yet used independently by the child. Corresponding toy sets were purchased for each book, and all materials were provided to families. Target words and themes for each child are listed in Supplementary File S1. Caregivers used their own internet-enabled device or a provided iPod Touch to record sessions.

### 2.8. Operational Definition and Dependent Variables

#### 2.8.1. Fidelity

Implementation fidelity, the primary caregiver dependent variable, was coded using checklists adapted from Kim et al. (2020) and McLeod et al. (2017). The reading criteria checklist contained 11 binary items (scored as a percentage of affirmative responses, maximum 100%) and three not-applicable items; the play criteria checklist contained seven items. Coaching fidelity was assessed against a separate mastery criteria checklist. Fidelity was calculated separately for reading, play, and total, yielding three measures. Fidelity was calculated as (a) reading fidelity, (b) play fidelity, and (c) composite fidelity. The criterion to withdraw coaching was at least 80% fidelity in both reading and play for two consecutive sessions with primary materials; it was thus possible to exceed 80% composite fidelity while still requiring coaching. Coaching fidelity was calculated using an eight-item checklist based on Akemoglu et al. (2022); scoring forms are in Supplementary File S2.

#### 2.8.2. Child Target Word Use

Unprompted target word use was selected as the primary child outcome because vocabulary is a core component of social communication for young autistic children, foundational to initiating and sustaining reciprocal verbal exchanges (American Psychiatric Association, 2013; Prelock & Nelson, 2012), and early vocabulary predicts later language and academic outcomes (Marchman & Fernald, 2008; Sénéchal & LeFevre, 2014). Target words were confirmed absent at intake, meaning gains represented genuine expansion of the functional communicative repertoire. Within the EMT framework, unprompted use indexes the child’s ability to communicate in natural contexts without adult direction (Kaiser & Hester, 1994; Kaiser et al., 2000) and measuring word use across reading and play evaluated whether gains extended beyond a single activity context (Hume et al., 2021; Kim et al., 2020; McLeod et al., 2017).

The child dependent variable was correct, unprompted target word use, defined as verbal use of a target word occurring spontaneously (Table 3), following a caregiver model, within three seconds of natural caregiver use, after a wait time prompt, or after an open-ended question prompt (Kim et al., 2020; McLeod et al., 2017). Word use after a choice question or mand model prompt was not considered unprompted. This definition follows McLeod et al. (2017) and Kim et al. (2020). As used here, “unprompted” reflects the EMT framework’s distinction between adult behaviors that supply the target word and those that create an opportunity for the child to generate it: time delays and open-ended questions do not include the word and require the child to initiate it independently; natural caregiver use allows the child to produce it in the next turn without direction. By contrast, choice questions and mand models supply the word and elicit repetition and are therefore excluded. While this captures a range of responding rather than purely spontaneous use, it is applied consistently in this line of research and is grounded in the EMT prompting hierarchy (Kim et al., 2020; McLeod et al., 2017). Session lengths ranged from 5 to 36 min (most 10–15 min). Rate per minute of unprompted target word use was calculated separately for reading, play, and total.

#### 2.8.3. Baseline

Each dyad received two theme sets (book, toys, target vocabulary), randomly assigned as primary or generalization targets. Caregivers read and played with their child as they typically would; sessions were recorded via Zoom and coded for child target word use and caregiver implementation fidelity. The length of the sessions varied based on the length of the book and the child’s engagement during play. The length of time spent reading during baseline varied by dyad with Lucy’s and Gianna’s time reading ranging from 5 min to 7 min, Mary’s and Elias’ time reading ranging from 5 min to 12 min, Theresa’s and Anne’s time reading ranging from eight minutes to 12 min, and Cheryl’s and Skyler’s time reading ranging from 5 min to 9 min. Lucy’s and Gianna’s time spent playing ranged from 10 min to 12 min, Mary’s and Elias’ time playing ranged from 1 min to 2 min, Theresa’s and Anne’s time playing ranged from 3 min to 8 min, and Cheryl’s and Skyler’s time playing ranged from 1 min to 8 min.

#### 2.8.4. Training

Following baseline, caregivers completed three one-hour instructional sessions via Zoom using modules adapted from Dillehay (2023). Instruction followed behavior skills training (BST) procedures (Schaefer & Andzik, 2021; Ward-Horner & Sturmey, 2012), including didactic instruction, modeling, role-play, and feedback on the shared book reading (Table 3) and EMT (Table 4) strategies. Caregivers were taught only with primary target materials. During training, caregivers were provided with checklists corresponding to the intervention fidelity criteria (see Supplementary File S4). 

#### 2.8.5. Implementation with Supported Coaching

Following instruction, caregivers implemented the intervention package with supported coaching. Sessions were recorded via Zoom, reviewed by the primary coder the following day, and coaching was provided within 24 h when feasible. Caregivers implemented the intervention two to three times per week and were instructed not to implement again until after coaching was received. Coaching procedures were adapted from Akemoglu et al. (2022). The coach met with the caregiver following each implementation session and began by greeting the caregiver and reviewing a segment of the session video. Most coaching sessions occurred within 24 h of the caregiver’s previous implementation. When meeting within 24 h was not feasible, caregivers were instructed to pause implementation until they could meet for coaching. The coach identified strengths in the caregiver’s implementation during both the reading and play portions, indicated one area of growth, and modeled the target skill. The coach and caregiver then practiced the skill through role-play, and the caregiver was given the opportunity to ask questions. The session concluded with the coach and caregiver collaboratively planning the next implementation and coaching session. Breaks in data collection for Cheryl and Skyler and Mary and Elias during this phase were due to unforeseen family circumstances. Caregivers were provided coaching until they reached the mastery criteria of 80% implementation fidelity for both play and reading for two consecutive sessions. Lucy completed four coaching sessions throughout the duration of the study with these coaching sessions ranging from 11 min to 15 min. Mary completed nine coaching sessions throughout the study with the coaching sessions ranging from 7 min to 24 min. Theresa completed five coaching sessions ranging from 8 min to 33 min. Cheryl completed seventeen coaching sessions ranging from 8 min to 18 min. Coaching sessions varied in length depending on number of caregiver questions and the amount of practice needed for the caregiver to show mastery of the skill from the coaching session.

During intervention, time spent reading for Lucy and Gianna ranged from 5 min to 12 min, Mary’s and Elias’ time spent reading ranged from 6 min to 16 min, Theresa’s and Anne’s time spent reading ranged from 5 min to 24 min, and Cheryl’s and Skyler’s time spent reading ranged from 4 to 19 min. During intervention, the time spent playing for Lucy and Gianna ranged from 5 min to 20 min, time spent playing for Mary and Elias ranged from 1 min to 10 min, time spent playing for Theresa and Anne ranged from 2 min to 10 min, and time spent playing for Cheryl and Skyler ranged from 2 min to 5 min.

#### 2.8.6. Independent Implementation

After meeting the coaching criterion, caregivers implemented the intervention independently. Implementation fidelity and child word use continued to be monitored; additional coaching was provided if fidelity fell below 80% for two consecutive sessions. Child mastery criteria were unprompted use of all target words at least once during reading and once during play for two consecutive sessions.

### 2.9. Generalization and Maintenance

Generalization probes assessed whether caregivers and children generalized the intervention to secondary materials without coaching. Primary and generalization materials were counterbalanced, and caregivers were naive to assignment. Probes alternated in a two-primary, one-generalization pattern across all phases, except for Lucy and Gianna who did not complete baseline generalization probes due to their short baseline. Maintenance probes were conducted four weeks after the intervention was withdrawn, with caregivers resuming implementation for five sessions using the same alternating schedule. No additional coaching or instruction was provided.

### 2.10. Interobserver Agreement

IOA was calculated for caregiver fidelity (reading and play) and child unprompted word use (reading and play) for at least 30% of sessions per phase. A graduate student in special education served as secondary coder, trained to at least 80% agreement via dual coding. The secondary coder was naïve to condition and used the same forms as the primary coder; reading-fidelity scores at or above 11 items were treated as equivalent for IOA. Coaching fidelity was dual-coded by a secondary coder with a PhD in special education and expertise in evidence-based coaching.

### 2.11. Social Validity

Social validity data were collected at the end of the study by sending participating caregivers a researcher-designed survey (see Supplementary File S3) which included a series of statements with a Likert scale rating system and a place for open-ended feedback. Caregivers were given the option to provide additional feedback through an interview. While the survey was sent to all caregivers, only two completed and returned it. No participants opted to provide additional feedback through an interview. These procedural limitations, specifically a 50% survey return rate and the absence of interview data, substantially constrain the interpretability of the social validity findings, and any conclusions drawn should be considered preliminary. Additionally, social validity was assessed using the framework of seven elements described by Reichow et al. (2008), considering three elements: the social meaningfulness of outcomes, the resources required of participants, and the ecological validity of the intervention. First, the primary child outcome, unprompted target word use, was evaluated for social meaningfulness; vocabulary acquisition is a common target in educational and therapeutic programming for autistic children (American Psychiatric Association, 2013), and gains in this area have direct relevance to children’s communicative development. Second, caregiver burden was considered in relation to the time and resources required for instruction and implementation; while individual coaching sessions were brief, the fully synchronous model created logistical demands that may affect feasibility in typical service delivery contexts. Third, ecological validity was assessed by considering whether the intervention setting and agent were natural to the child’s daily routines; sessions took place in families’ homes and caregivers served as the intervention agents, both of which support the ecological validity of the package.

### 2.12. Analysis

Rate per minute of child unprompted target word use was graphed across phases for reading, play, and total; the total graph served as the primary basis for determining functional relations. Data were analyzed using visual analysis procedures outlined in Kratochwill et al. (2010), examining changes in trend, level, and variability across phases. Consistent with these standards, six features were evaluated: level (mean or median within a phase), trend (direction and rate of change), variability (fluctuation around the trend line), immediacy of effect (speed and magnitude of change at phase transitions), data overlap (proportion of data points in adjacent phases sharing the same range), and consistency across tiers (replication of the pattern following intervention introduction across participants). A functional relation was identified when the data showed a predictable and replicable change temporally associated with the intervention across at least three tiers, with attention to whether pre-existing baseline trends could account for observed changes (Kratochwill et al., 2010; Ledford & Gast, 2018). Effect size estimates for caregiver implementation fidelity were calculated using both standard mean difference (SMD) (Hedges et al., 2012) and Tau-U (Parker et al., 2011). Effect sizes for child unprompted word use were calculated using Tau-U only; SMD was not appropriate for these variables given the zero and near-zero levels of responding in baseline. Effect sizes were computed via the online calculator from Pustejovsky et al. (2024). Effect size summaries are available in table form in Supplementary File S1.

## 3. Results

**Figure 1:**
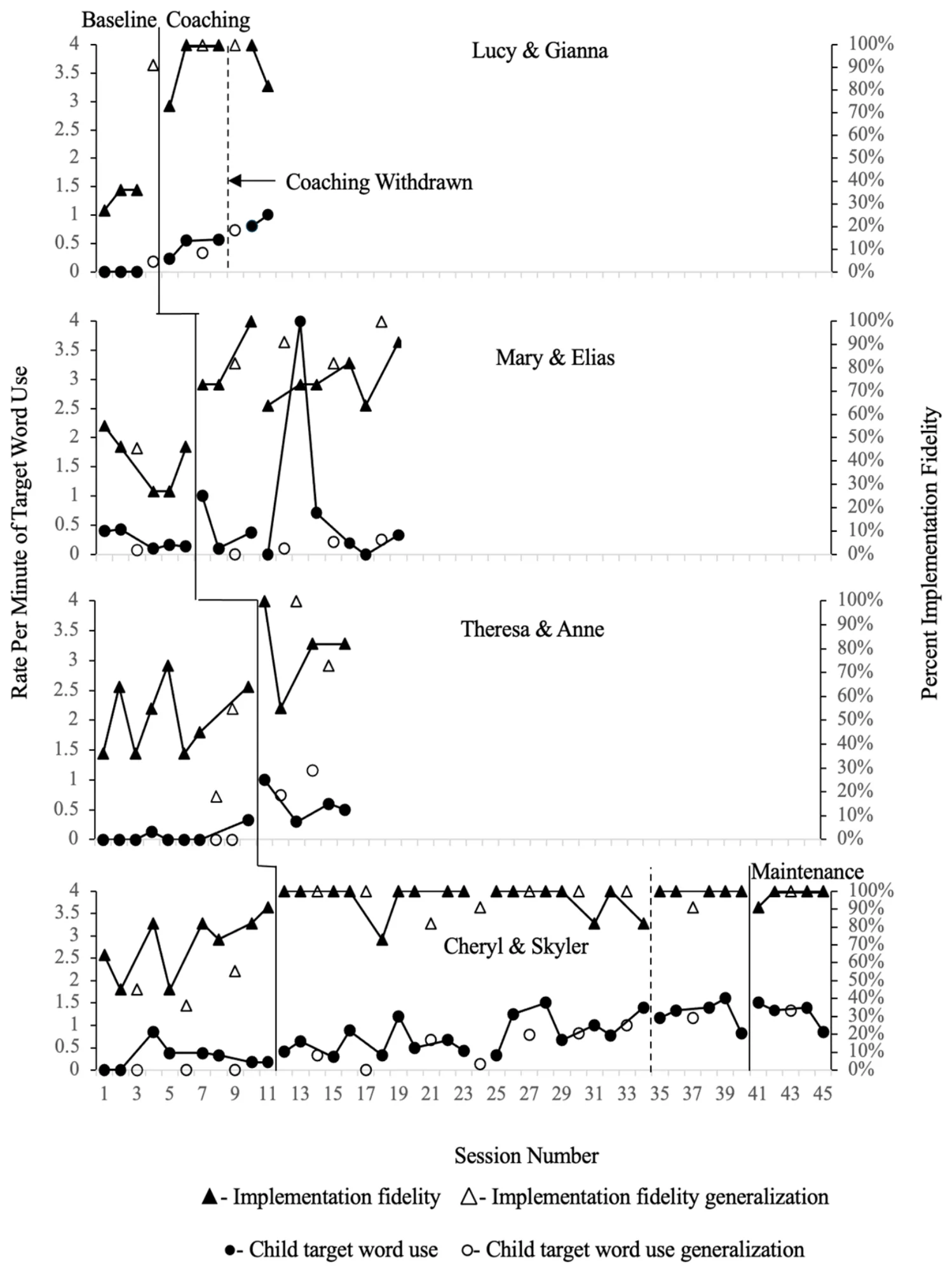
Rate of target word use (left axis, circle) and caregiver implementation fidelity (right axis, triangles) during reading.

**Figure 2:**
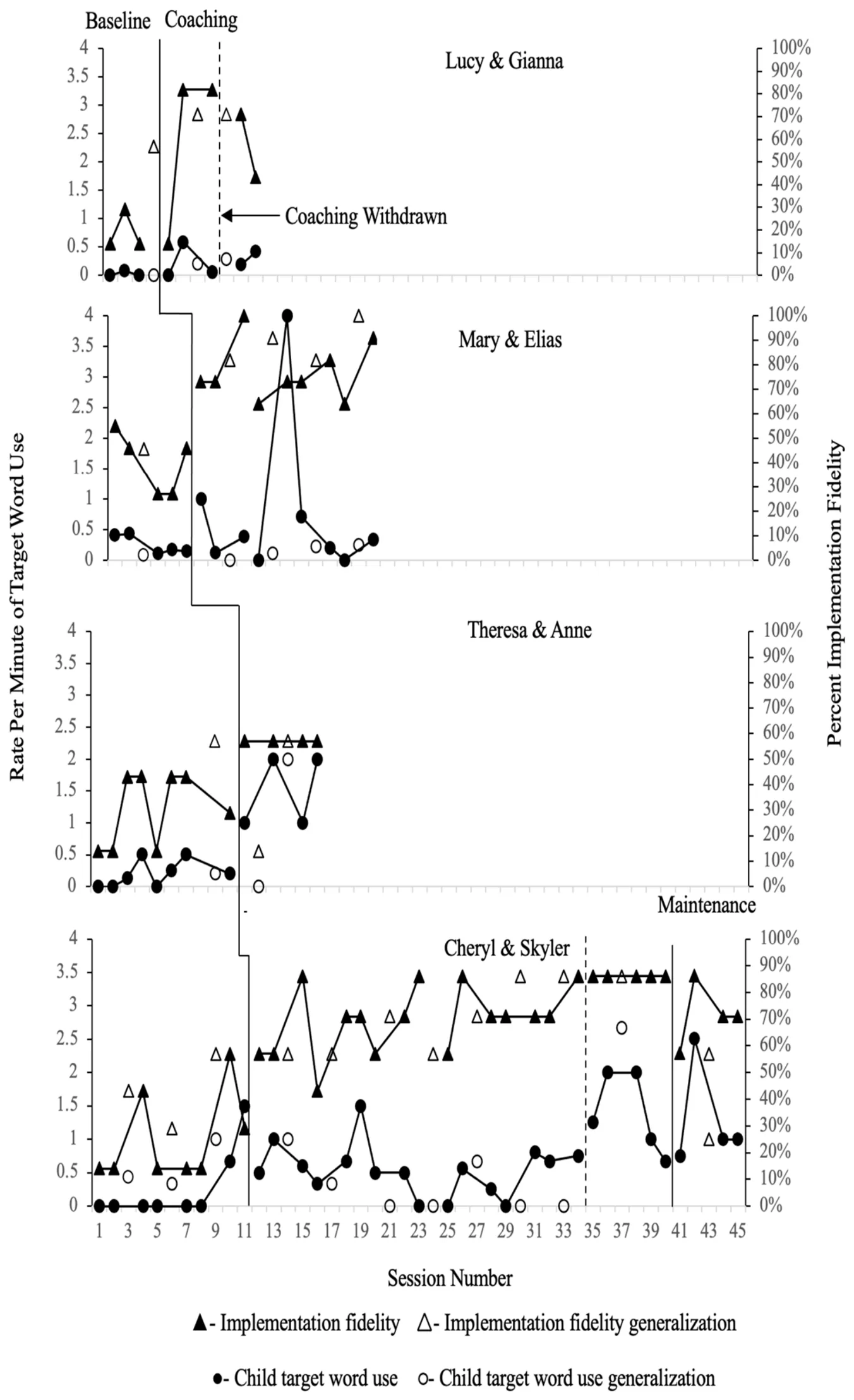
Rate of target word use (left axis, circles) and caregiver implementation fidelity (right axis, triangles) during play.

**Figure 3:**
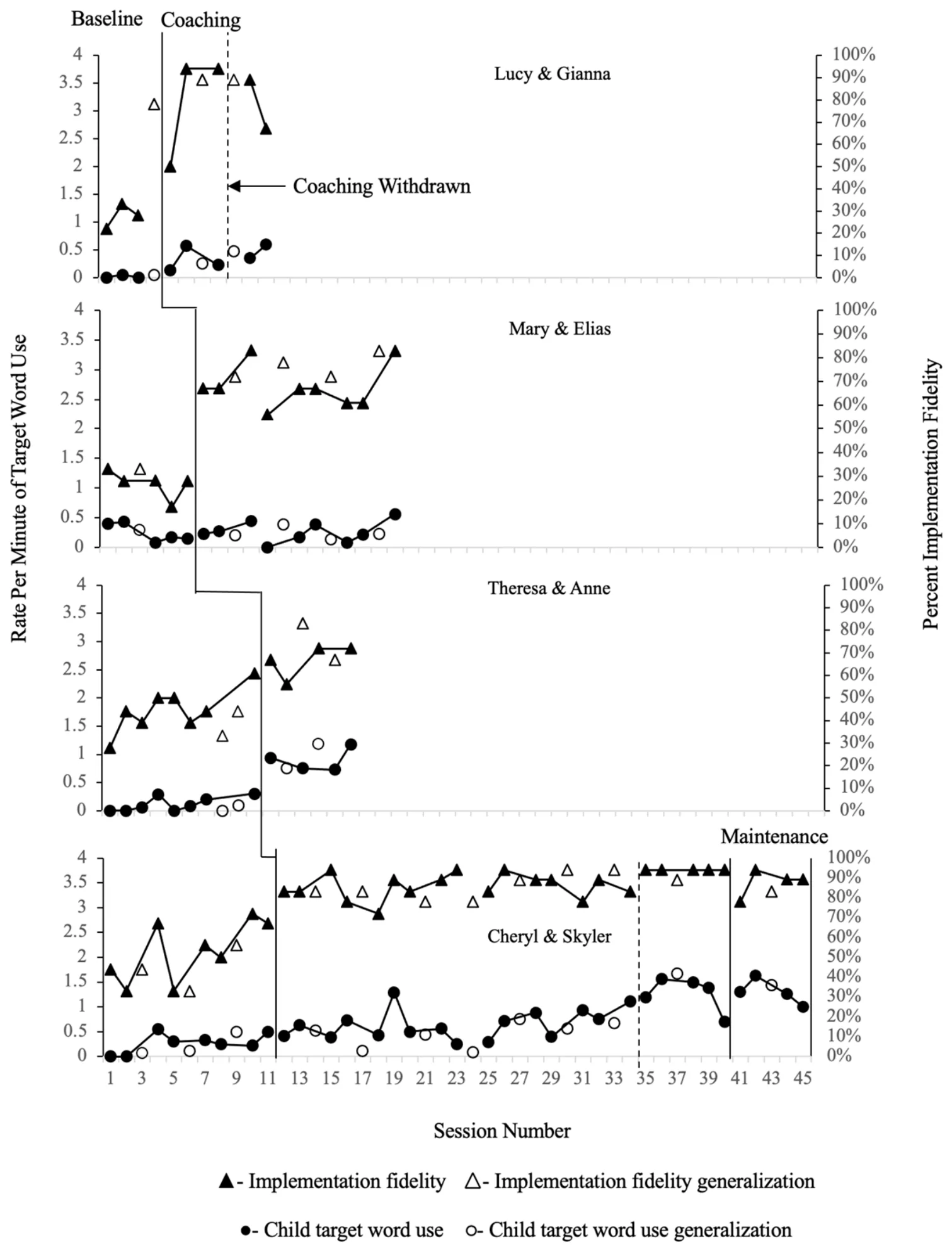
Overall rate of target word use (left axis, circles) and caregiver implementation fidelity (right axis, triangles) with book reading and EMT combined.

Results for levels of caregiver fidelity and the children’s rate per minute of target word use are presented graphically during reading (Figure 1), play (Figure 2), and in total (Figure 3). Across all four dyads, caregiver implementation fidelity increased following training and coaching, with some evidence of generalization to untrained materials and routines for caregivers who remained in the study, though generalization sequences were not completed by all dyads. Two caregivers reached mastery criteria and completed maintenance probes, while two withdrew prior to mastery due to family circumstances. Visual analysis demonstrated a functional relation between coaching and caregiver implementation fidelity. Child outcomes were more variable, though three of four children demonstrated increases with variable response patterns in unprompted target word use following intervention, with effects during shared book reading generally stronger than during play, though this pattern was not uniform across participants. For one child, Elias, effects were limited, with Tau-U values of 0.21 for reading and total and insufficient baseline data to calculate play effect sizes; these results do not constitute clear evidence of intervention impact. For the remaining children, effects were variable in strength and in some cases limited to reading rather than play. Generalization of child word use was observed across most dyads, though with variable strength across participants and measures, and maintenance data from completed cases suggested that gains were largely maintained following withdrawal of coaching and intervention supports, though maintenance was assessed only for Cheryl and Skyler and cannot be generalized to the full sample. Descriptive statistics for caregiver implementation fidelity and child target word use across phases are presented in Table 5 and Table 6, respectively.

### 3.1. Lucy and Gianna

During baseline, Lucy’s implementation fidelity was consistently low across all components. Her fidelity in reading never exceeded 40%; play: never exceeded 29%; total: never exceeded 36%. Following training, Lucy reached criterion in reading within two sessions and in play within three (coaching fidelity: *M* = 100%). Total fidelity showed a clear change in level and increasing trend, reaching 94% for two consecutive sessions. After coaching was withdrawn, fidelity declined after session 10 in reading and dropped below 80% in play; additional coaching was offered, but Lucy subsequently withdrew due to family circumstances. Generalization probes reflected improved fidelity, though slightly below target probe levels. Lucy demonstrated increases in fidelity across reading (SMD = 6.63, 95% CI: [2.78, 10.48], Tau-U = 0.92), play (SMD = 2.80, 95% CI: [0.80, 4.79], Tau-U = 0.83), and total (SMD = 2.36, 95% CI: [1.25, 3.46], Tau-U = 0.96). Gianna showed a consistent rate of zero unprompted word uses per minute in baseline across all three measures. Following the introduction of the intervention, a clear change in level emerged across reading, play, and total, with an increasing trend in reading from session five onward, a peak of 0.57 uses per minute at session five in total, and a peak in play at session six after an initially low level of responding. These gains were maintained after coaching was withdrawn, and generalization probes showed a similar increase. Gianna demonstrated increases in word use during reading (Tau-U = 1.00), play (Tau-U = 0.58), and total (Tau-U = 0.96).

### 3.2. Mary and Elias

During baseline, Mary’s implementation fidelity was consistently low across all components (reading: *M* = 41%, range = 27–55%; total: *M* = 28%, range = 17–33%); only one play baseline session was available due to Elias withdrawing assent (*M* = 22%, range = 14–29%). Following a two-week gap due to family travel, a booster training session was provided before intervention began. With coaching (*M* = 99%, range = 88–100%), fidelity increased substantially in reading (*M* = 81%, range = 64–100%) and total (*M* = 71%, range = 61–83%), though the trend remained highly variable and Mary did not reach criterion in any component. Generalization probes exceeded baseline in reading and total and exceeded target probes in play. Mary demonstrated increases in reading (SMD = 2.89, 95% CI: [1.17, 4.60], Tau-U = 1.09) and total fidelity (SMD = 12.30, 95% CI: [−1.19, 25.80], Tau-U = 1.08). Effect sizes for play could not be calculated due to insufficient baseline probes, and with only one available play baseline session, conclusions about Mary’s play fidelity cannot be adequately supported by the data. This should be considered a significant constraint on the interpretation of play fidelity outcomes for this dyad. Elias’s baseline word use was consistently low in reading (*M* = 2.89, range = 0.08–0.43) and total (*M* = 0.25, range = 0.08–0.43); only two play probes were available (*M* = 0.75, range = 0–1.5). Following the introduction of the intervention, reading responding was variable (*M* = 0.56, range = 0–0.71) with a notable peak of four uses per minute at session 14, and a corresponding increase alongside improved caregiver fidelity was observed from sessions 17 through 19. In play, Elias showed an immediate increase (*M* = 3.39, range = 0–2) before declining with a corresponding drop in caregiver fidelity; total word use remained low overall (*M* = 0.25, range = 0–0.56) with a slight increasing trend from session 16 onward. The dyad withdrew after 19 sessions due to extenuating circumstances. Play generalization probes were consistently lower than target probes. Total generalization probes showed a similar rate. Tau-U could not be calculated for play. Elias demonstrated modest increases in word use during reading and total (Tau-U = 0.21 for both); however, Tau-U values of this magnitude indicate limited intervention effects, and the absence of calculable play effect sizes further restricts interpretation. Overall, child outcome effects for Elias were limited and should not be characterized as clear evidence of intervention impact.

### 3.3. Theresa and Anne

During baseline, Theresa showed variable fidelity across all components, with an increasing trend established in the final three sessions in reading (*M* = 44%, range = 18–73%) and in total (*M* = 39%, range = 28–61%); play never exceeded 50% (*M* = 33%, range = 14–57%). Following training, fidelity was higher throughout the coaching phase across all components, reading (*M* = 93%, range = 55–100%), play (*M* = 51%, range = 14–57%), and total (*M* = 70%, range = 56–83%), with coaching fidelity consistent throughout (*M* = 100%). Two gaps between sessions exceeding two weeks likely contributed to variability. Theresa did not reach criterion in any component before being discharged due to family circumstances. Generalization probes showed a similar increasing trend in reading and total, with mixed patterns in play. Theresa demonstrated increases in fidelity in reading (SMD = 1.83, 95% CI: [0.62, 3.05], Tau-U = 0.80), play (SMD = 0.94, 95% CI: [−0.12, 2.00], Tau-U = 0.39), and total (SMD = 2.58, 95% CI: [1.17, 3.99], Tau-U = 0.77). Anne’s baseline word use was consistently low and near-zero in reading (*M* = 0.04, range = 0–0.33) with a slight increasing trend at the end of baseline; Play use never exceeded one use per minute, and total use showed a low level with an increasing trend in the final sessions (*M* = 0.09, range = 0–0.29). Following the introduction of the intervention, a clear change in level emerged across all three measures: reading (*M* = 0.72, range = 0.3–1.16), play (consistently at or above one use per minute), and total (*M* = 0.92, range = 0.73–1.18). However, a decrease in reading at the 13th probe corresponded to a drop in caregiver fidelity, and a clear trend could not be established in play. The dyad was discharged before Anne reached mastery criteria, and these results should be characterized as preliminary. Because Theresa did not reach criterion in any fidelity component and the dyad was discharged before Anne reached child mastery criteria, the apparent changes in level cannot be interpreted as evidence of a complete or stable intervention effect. Generalization was mixed in play but showed a similar increase from baseline in reading and total. Anne demonstrated increases in word use in reading (Tau-U = 0.85), play (Tau-U = 0.62), and total (Tau-U = 0.68); however, given that the dyad was discharged before mastery criteria were reached, these effect sizes should be interpreted cautiously as reflecting an incomplete rather than a concluded intervention trajectory.

### 3.4. Cheryl and Skyler

During baseline, Cheryl showed variable fidelity with an increasing trend toward the end across all components (reading: *M* = 58%, range = 36–91%; play: *M* = 30%, range = 14–57%; total: *M* = 46%, range = 33–72%). Following training, Cheryl reached criterion in reading within two sessions; coaching fidelity was high throughout (*M* = 98%, range = 88–100%). Reading fidelity remained high and stable for the duration, dipping below 80% at only one probe (*M* = 93%, range = 73–100%). A clear increasing trend in play was not established until after session 32, and Cheryl reached criterion for play after session 38. Total fidelity showed a clear positive change in level (*M* = 90%, range = 78–94%). Cheryl maintained high fidelity in the immediate maintenance phase (total: 100%; reading: *M* = 98%, range = 91–100%). In the delayed maintenance phase, play fidelity showed a considerable drop before recovering to above baseline (*M* = 68%, range = 57–86%), and total initially dropped to 78% before recovering to at or above 89% (*M* = 87%, range = 78–94%). Generalization data showed high and stable patterns in reading and a clear change in level in total. Cheryl demonstrated increases in fidelity in reading (SMD = 1.58, 95% CI: [0.73, 2.42], Tau-U = 0.88), play (SMD = 2.26, 95% CI: [1.13, 3.40], Tau-U = 0.94), and total (SMD = 2.36, 95% CI: [1.25, 3.46], Tau-U = 0.92). During baseline, Skyler showed a consistently low rate of word use in reading (*M* = 0.19, range = 0–0.86) and a variable total rate with no established trend (*M* = 0.24, range = 0–0.55); in play, the rate was zero for the first six sessions before a considerable increasing trend through the end of baseline (*M* = 0.36, range = 0–1.5). This pre-existing increasing trend in play word use during baseline complicates causal attribution of later play gains to the intervention and play outcomes for Skyler should be interpreted with particular caution. Following the introduction of the intervention, reading word use was variable but trended upward throughout (*M* = 0.84, range = 0–1.6); play responding showed initial decreases and variability before a clearly increasing trend emerged after session 29 (*M* = 0.72, range = 0–2.67); and total responding showed no discernible change in level early on (*M* = 0.77, range = 0.09–1.67) before a clear increasing trend from session 25. Generalization probes showed a similar increasing trend in reading. During immediate maintenance, gains were initially maintained before declining; in the delayed maintenance phase, play (*M* = 1.25, range = 0.75–2.5) and total (*M* = 1.1, range = 1–1.63) recovered to above baseline despite a decreasing trend, while reading remained stable (*M* = 1.28, range = 0.86–1.5). Skyler reached mastery after the first two delayed maintenance probes. Skyler demonstrated increases in word use in reading (Tau-U = 0.73), play (Tau-U = 0.27), and total (Tau-U = 0.72). The notably weaker effect size for play (Tau-U = 0.27), in the context of the pre-existing baseline trend documented above, means that play gains cannot be confidently attributed to the intervention. Reading and total effects were stronger and less confounded by baseline trends.

Two effect size reporting issues warrant explanation. First, Tau-U values exceeding 1.0 for Mary in reading (Tau-U = 1.09) and total (Tau-U = 1.08) reflect baseline correction: when baseline shows a decreasing trend, the correction adjusts the statistic upward, producing values outside the typical 0 to 1 range (Parker et al., 2011). These should be interpreted as indicating strong positive change relative to the baseline trend, not as an effect exceeding the scale. Second, SMD confidence intervals were wide or included negative values for Mary’s total fidelity (SMD = 12.30, 95% CI: [−1.19, 25.80]) and Theresa’s play (SMD = 0.94, 95% CI: [−0.12, 2.00]), reflecting small phase sample sizes and high variability; intervals crossing zero indicate the effect cannot be distinguished from zero with confidence. Visual analysis provides the more reliable basis for interpreting these findings.

### 3.5. Interobserver Agreement

Interobserver agreement (IOA) for caregiver mastery of the training was calculated by reviewing the last day of training with a mastery checklist of intervention strategies using total IOA (*M* = 86.5%, range = 76–100%). A summary of IOA for all participants can be found in Table 7.

Although mean IOA values were at or above 80% for all measures across dyads (see Table 7), the ranges indicate that agreement was substantially lower in individual sessions, and these values should be interpreted with caution. For Cheryl and Skyler, play fidelity IOA ranged from 20% to 100%, with one session at 20% during baseline and one at 40% during intervention; reading fidelity IOA also showed notable variability, ranging from 40% to 100%, with multiple sessions below 75% across both phases. These low IOA values reduce confidence in the precision of Cheryl’s fidelity data, particularly for play, despite this dyad producing the strongest overall fidelity outcomes. For Mary and Elias, reading fidelity IOA included one session at 43% during the intervention phase, which, combined with the already-variable fidelity trend documented for Mary, further limits confidence in the reading fidelity data for this dyad. For Theresa and Anne, child word use during reading showed IOA values of 50% to 75% across multiple sessions in both baseline and intervention phases, which reduces confidence in the precision of Anne’s reading word use data and should be considered when interpreting the apparent changes in level reported above. For Lucy and Gianna, child word use during play included one session at 40%, and play fidelity included one session at 50% during baseline. Across all dyads, word use IOA was generally more variable than fidelity IOA, likely reflecting the difficulty of reliably coding low-frequency verbal behavior, and session-level values should be considered when interpreting findings.

### 3.6. Social Validity

Social validity data were collected via a researcher-designed survey (see Supplementary File S3) sent to all caregivers at study completion; two returned completed surveys, and no participants opted for an interview. Cheryl indicated that the intervention was worth her time and that Skyler benefited from it. In the open-ended feedback, Cheryl described Skyler as having a “language explosion,” noting that his speech therapist commented on how much more he was talking. She also said she felt comfortable discussing anxieties with the coach and that feedback was consistently positive. Outside of the survey, Lucy noted that Gianna had begun asking her to play, something that had not occurred before the intervention. These reports reflect the experiences of one dyad who completed the full protocol and one who withdrew after reaching mastery criteria and may not be representative of the range of caregiver experiences across the study. Social validity was also evaluated using the framework described by Reichow et al. (2008), though the following conclusions should be interpreted cautiously given the limited evidence base. Outcomes were socially meaningful: unprompted vocabulary use is a direct target in both educational and therapeutic programming for autistic children, and most children showed increases in target word use from baseline, though the strength of evidence varied across participants, with more modest effects observed for some children and pre-existing baseline trends complicating causal interpretation for others. Based on the two survey responses received, both from caregivers who completed the protocol, caregiver burden appeared moderate, though caregivers who withdrew did not return surveys and may have experienced greater burden, limiting conclusions about acceptability across the full sample. These findings should not be interpreted as evidence of strong social acceptability. The home-based, caregiver-implemented design supports ecological validity, though broader conclusions about social acceptability await more complete evidence.

## 4. Discussion

This novel study evaluated caregiver-implemented EMT with shared book reading through telepractice training and coaching, using BST as the training framework (Schaefer & Andzik, 2021; Ward-Horner & Sturmey, 2012). Two hypotheses were tested: that coaching would produce a functional relation with caregiver fidelity, and that caregiver implementation would produce a functional relation with child target vocabulary use. A functional relation was demonstrated for caregiver implementation fidelity; however, evidence for child target word use was insufficient to establish a clear functional relation, given the variable outcomes, incomplete tiers, baseline trends, and limited effects for some children. Three of the four child participants demonstrated an increase in unprompted target word use; however, these gains were variable across the duration of the study, and one child, Elias, showed variable responding not significantly different from baseline. Skyler demonstrated a considerable increasing baseline trend in play, complicating causal attribution of play gains to the intervention, and Anne was discharged before reaching mastery criteria. Taken together, these patterns indicate that the evidence for child target word use is promising but variable and should be interpreted with caution. This finding contrasts somewhat from prior studies using this intervention package implemented by trained researchers rather than caregivers (Kim et al., 2020; McLeod et al., 2017). The clinical significance of the child outcome changes warrants consideration. Unprompted word use reflects self-initiated communication rather than directed repetition, consistent with EMT’s functional communication goals (Kaiser & Hester, 1994), and because target words were absent from each child’s productive vocabulary at intake, gains represent genuine repertoire expansion rather than practice effects. There was also anecdotal evidence of change beyond sessions: Cheryl reported Skyler experienced a “language explosion” and that his speech therapist noted increased communication. However, word use was measured only within structured sessions using specific materials, so whether gains generalized to everyday communication cannot be confirmed from the present data; future studies should include broader language measures to evaluate functional significance.

Promisingly, all caregivers showed a clear increase in implementation fidelity from baseline following training and coaching. However, only two of four caregivers reached the mastery criterion, and maintenance data were consequently limited to those two dyads, which constrains conclusions about full mastery and the durability of intervention gains. The consistency of fidelity gains across dyads should not be taken to imply a uniform process, given the substantial heterogeneity of the sample. Children ranged from 3 to 9 years of age and differed markedly in language profiles and educational contexts; factors such as child age, existing communicative repertoire, and setting may each have influenced outcomes independently. Individual dyad-level results, rather than cross-dyad summaries, should be treated as the primary unit of interpretation. Caregivers consistently reached fidelity criteria for reading more quickly than for play, and in some instances implemented reading at 100% fidelity while play lagged behind. Several factors likely account for this pattern. First, the reading checklist contained 13 items compared to seven for play, meaning that missing just two play items resulted in fidelity below 80%. Second, shared book reading is more structured than free play (Clemens & Kegel, 2020; Noble et al., 2019), which may have made adherence to reading criteria more formulaic. Third, play-based EMT required more responsive, moment-to-moment decision-making. Book reading provides predictable narrative cues for when to model and prompt, whereas play requires caregivers to follow the child’s lead in an open-ended context, selecting and timing prompts in response to rapidly changing child actions (Kaiser & Hester, 1994; Schreibman et al., 2015). When children shift play routines unexpectedly, caregivers must judge in real time which target words to embed naturally, a demand structured training alone may not fully address (McLeod et al., 2017; Vivanti & Zhong, 2020), which may explain why play fidelity remained lower and more variable. Moreover, the caregivers improving fidelity while not always meeting target thresholds is consistent with the current literature on caregiver-mediated NDBI (van Noorden et al., 2026). Baseline fidelity was not uniformly stable. Theresa showed an increasing trend in reading and total fidelity across the final three baseline sessions, and Cheryl showed an increasing trend toward the end of baseline across all components. Pre-existing trends reduce confidence in the functional relation claim for those dyads, as post-intervention gains may partly reflect continuation of a pre-existing trajectory (Kratochwill et al., 2010; Ledford & Gast, 2018), and are most relevant for Theresa and, to a lesser extent, Cheryl.

The findings must be considered in light of the feasibility challenges that emerged. Although the intervention was designed with a telepractice model specifically to maximize flexibility for families (Pacione, 2022), two dyads exited before reaching mastery criteria. It is important to distinguish the nature of attrition. Lucy withdrew after already reaching mastery criteria, reflecting external family circumstances rather than a failure to demonstrate the intervention skills. Mary and Theresa withdrew during coaching without reaching criterion; both experienced multi-week data collection disruptions (family travel for Mary; session gaps exceeding two weeks for Theresa), and it is not possible to fully disentangle whether withdrawal reflected external circumstances, the demands of the synchronous model, or both. This may indicate that the 80% play fidelity criterion was difficult to achieve within the synchronous model, or that additional supports, such as annotated video models or individualized implementation checklists, are needed. The fully synchronous design also created a scheduling burden. Training required three 1 h blocks and each coaching session required a 15 min virtual meeting coordinated across both schedules. An asynchronous or hybrid coaching model could reduce this burden while preserving rigor (Akemoglu et al., 2022; Pacione, 2022). More broadly, the intervention demands may have exceeded what is realistic for many families. Caregivers completed three 1 h training sessions, recorded sessions multiple times per week, and participated in synchronous coaching within 24 h, a substantial commitment alongside existing caregiving responsibilities. The study provides preliminary efficacy evidence for caregiver fidelity, but attrition, incomplete mastery, and scheduling burden indicate the model was not fully feasible for the majority of the sample. Telepractice reduces logistical barriers but does not resolve the demands of a synchronous protocol. Despite attrition, most caregivers generalized fidelity to untrained materials, though child word use generalization was more variable and limited for some. It is also unclear whether gains in target word use reflected genuine vocabulary acquisition, contextual practice effects, or increased session opportunities, as word use was measured only within structured sessions; future research should include language samples or caregiver report.

Session duration varied substantially across dyads and phases, with play sessions particularly short for some dyads; during baseline, play sessions for Mary and Elias ranged from 1 to 2 min, and for Cheryl and Skyler from 1 to 8 min. When sessions are very short, rate-per-minute estimates become more sensitive to individual utterances, and very short play sessions may have provided insufficient opportunity for target word use, making it difficult to distinguish true absence of word use from sessions in which opportunities simply did not arise. This limits the interpretability of play word use data for dyads with very short sessions and should be considered when evaluating play outcomes.

This study extends prior research in three ways. First, it builds on the EMT literature base with shared book reading (Kim et al., 2020; McLeod et al., 2017) and demonstrated a treatment effect on caregiver implementation fidelity when the intervention is implemented by caregivers rather than trained researchers or clinicians: in both prior studies using this intervention package, the intervention was delivered by a trained researcher or clinician directly with the child, and neither evaluated caregiver training or coaching as the mechanism of delivery. The present study is therefore the first replication of the EMT with shared book reading package in a caregiver-mediated format, though evidence for child target word use was mixed across participants and should be interpreted with caution given the variable response patterns observed. This finding is a meaningful step toward real-world applicability, though the feasibility concerns noted above indicate that the fully synchronous coaching model warrants further development. Second, it contributes to the line of work on caregiver coaching for naturalistic interventions and NDBIs by demonstrating that caregivers can be coached toward acceptable implementation fidelity, though full mastery of the intervention package was achieved by only two of four caregivers, and evidence for maintenance is accordingly limited to those cases. It should also be noted that because the intervention included multiple components, including BST-based training, synchronous coaching, shared book reading procedures, EMT strategies, and interest-based material selection, the present design does not permit conclusions about which elements were responsible for the observed changes in caregiver fidelity or child word use. This represents a constraint on interpretation that applies across all three contributions noted above. Third, it contributes to the growing literature on telepractice-based coaching for NDBI (Ferguson et al., 2022; Meadan et al., 2016) and naturalistic book reading interventions (Akemoglu et al., 2022). The Zoom-based session upload model allowed families to implement at convenient times. Only coaching and training sessions required synchronous coordination, providing flexibility not always available with in-person delivery. However, the synchronous components, specifically training sessions and post-session coaching meetings, were most closely associated with scheduling burden and attrition. The upload flexibility for implementation sessions did not offset those demands, and the delivery medium should not be taken as evidence of overall feasibility. This study also extends feasibility with the materials used. Whereas prior studies used books created specifically for the intervention, this study used preexisting, interest-matched books, which may be more practical for service providers and offers greater flexibility through widely available library and lending resources (Kim et al., 2020; McLeod et al., 2017).

### 4.1. Limitations

Several limitations should be noted. The NCMBL design (Watson & Workman, 1981) offers weaker internal validity than a concurrent design, although as described in the Section 2, several design features were employed to minimize the primary threats identified for this design variation (Slocum et al., 2022a, 2022b), including independent home settings with no participant contact, randomly assigned and varied baseline lengths across tiers, and individualized target materials that precluded shared stimulus confounds (Ledford & Gast, 2018). Nonetheless, the weaker internal validity of the NCMBL relative to a concurrent design remains a constraint on causal inference and should be considered when interpreting the findings. Departures from the original protocol, including unplanned data collection breaks and breaks between reading and play for some participants, also limit interpretation, though both reflect the practical reality of attending to individual child and family needs. Attrition is a third and substantial limitation. Two dyads (Mary and Elias; Theresa and Anne) withdrew during coaching without reaching mastery, and a third (Lucy and Gianna) withdrew during independent implementation after mastery had been achieved. Withdrawal was attributed to family circumstances in all cases, though the contribution of the synchronous model’s demands cannot be fully separated. The loss of two uncompleted tiers weakens the replication logic that supports causal inference in multiple baseline designs (Ledford & Gast, 2018; Kratochwill et al., 2010), compounding the weaker internal validity of the NCMBL design. Maintenance data were collected only for Cheryl and Skyler. Furthermore, the sample was small and highly heterogeneous. Children ranged from 3 to 9 years of age, attended four different educational placements, and showed markedly different baseline language profiles; SCQ scores ranged from 4 to 13 and CDI word use percentile scores from the 10th to the 99th percentile (see Table 2). This degree of heterogeneity means that patterns observed across dyads may reflect different underlying processes rather than a common intervention effect, and aggregated descriptions risk obscuring meaningful between-participant differences. Findings should be interpreted at the level of the individual dyad, and replication with larger, more clearly defined samples is needed before drawing conclusions about for whom this intervention is most effective. Finally, because Elias withdrew assent during play, only two play baseline probes were available, limiting conclusions about intervention effects for that component. Measurement reliability is an additional limitation. IOA ranges included sessions as low as 20%, and low IOA was not confined to a single dyad or phase; the most notable concerns are play fidelity IOA for Cheryl and Skyler, reading fidelity IOA for Mary and Elias, and child word use reading IOA for Theresa and Anne, where multiple sessions fell below acceptable thresholds. The social validity measure is also constrained: the researcher-designed survey was unvalidated with unknown psychometric properties, and conclusions are limited by both the low response rate and the informal measure. Eligibility relied on the SCQ and CDI, which provide screening data but not a comprehensive diagnostic profile, limiting conclusions about for whom the intervention is most effective. Additionally, one child participant did not have a confirmed autism diagnosis at the time of enrollment; autism was suspected and an evaluation was in progress. This difference in diagnostic status should be considered when interpreting findings for this dyad, and study findings should not be interpreted as applying uniformly to children with independently confirmed autism diagnoses. The multi-component package did not include a component analysis, so it is not possible to determine which elements drove the observed outcomes; future research using dismantling designs would help identify active elements. Baseline instability is a further limitation: increasing fidelity trends for Theresa and Cheryl and increasing child word use trends for Anne and Skyler make it harder to attribute post-intervention changes to the intervention (Kratochwill et al., 2010; Ledford & Gast, 2018), and functional relation claims for these dyads should be interpreted with caution. Session duration variability also limits interpretation: play sessions as short as 1 to 2 min may not have provided adequate word use opportunity, and rate-per-minute estimates from very short sessions are susceptible to floor effects; future studies should standardize minimum session lengths.

### 4.2. Implications for Practice and Future Research

Although child outcomes were variable, caregivers consistently increased their use of naturalistic intervention strategies following training and coaching, supporting the use of BST as a caregiver training method and telepractice as a viable coaching medium, though these findings should be interpreted as demonstrating promise rather than established effectiveness, given that only two of four caregivers reached mastery criteria and maintenance evidence is limited accordingly. This intervention should be characterized as promising but requiring further development before broad implementation can be recommended. Modifications to reduce scheduling burden, such as hybrid or asynchronous coaching, are likely necessary but have not yet been evaluated; practitioner uptake should await evidence from a more feasible delivery model better aligned with the practical realities of families in early intervention.

Future research should evaluate asynchronous or hybrid coaching models to determine whether increased flexibility improves feasibility while maintaining intervention effects. Cascading coaching models (e.g., Meadan et al., 2020), in which researchers train service providers who in turn train caregivers, should also be examined, as they may support wider adoption and better reflect real-world service delivery. Additional work is needed to evaluate generalization across caregivers and settings. Future research should also use more varied social validity methods, including naïve raters, qualitative and mixed methods approaches, and strategies that better capture child perspectives. Studies should directly compare synchronous, asynchronous, and hybrid coaching formats to determine which best balances feasibility and fidelity (Akemoglu et al., 2022; Pacione, 2022). Replication with larger samples is needed, with greater baseline stability, independent diagnostic confirmation beyond screening tools such as the SCQ, and inclusion of families with greater cultural, linguistic, and socioeconomic diversity. Standardized language measures, such as language samples or norm-referenced vocabulary assessments, should supplement session-based outcomes to evaluate whether gains generalize to everyday communication. Finally, component analysis designs are needed to examine the relative contribution of individual strategies and to determine which strategy or combination of strategies are needed to produce an effect.

## Figures and Tables

**Table 1:** Participant Demographic Information.

Dyad	Caregiver Age	Child Age	Caregiver Race	Child Race	Caregiver Education Level	Child Educational Placement
Lucy & Gianna	43 years	3 years	Black	Black	Bachelor’s degree	Full-time applied behavior analysis clinic
Mary & Elias	45 years	5 years	Asian	Asian	Associate’s degree	Public school
Theresa & Anne	46 years	7 years	Black	Black	Bachelor’s degree	Public school
Cheryl & Skyler	42 years	9 years	Black	Black	Master’s degree	Specialist private school

**Table 2:** Assessment Information.

Child	SCQ Score	MacArthur Bates CDI Word Use Score	MacArthur Bates Word Forms Score	MacArthur Bates Word Endings Score	MacArthur Bates Complexity Score
Gianna	13	10th percentile	5th percentile	30th percentile	20th percentile
Elias	4	99th percentile	99th percentile	25th percentile	99th percentile
Anne	10	75th percentile	10th percentile	50th percentile	25th percentile
Skyler	9	80th percentile	10th percentile	30th percentile	20th percentile

**Table 3:** Enhanced Milieu Teaching Strategies.

Strategy	Definition	Example	Non-Example
Time-delay	The caregiver either places the desired item within sight but out of reach, or creates a scenario where the desired item is missing. Following creating the opportunity, the caregiver waits 3 s to give the child an opportunity to respond.	The caregiver holds a toy car in front of the child and waits three seconds for the child to request the item. The caregiver sets out toys for a tea party, and they withhold the teacups. They wait three seconds for the child to request the missing item.	Caregiver removes target item from line of sight and waits three seconds. Caregiver holds the item in front of the child and immediately give the child the item without a waiting period.
Open-ended question	The caregiver asks a question related to the target word that does not include the target word.	“What do we need to race?” “What’s missing from the kitchen?”	For the target word boat, the caregiver may say “where is the boat” Yes or no questions—“Is this a boat?”
Choice question	The caregiver asks a question for the child that includes choosing between two items. The caregiver uses the target word in the question.	“Do you want the car or the boat?” “Is the whale swimming or walking?”	Uses a choice question that does not include the target word such as “Do you want the whale or fish?” when the target word is boat. Exclude yes or no questions.
Mand model	The caregiver instructs the child to say the target word.	“Say race car.” “Say swimming.”	Caregiver labels the item without giving directive to the child to say the target word.
Use of wait time to promote communicative turn taking	The caregiver provides opportunities for the child to speak following caregiver speech. This involves pausing for a minimum of three seconds after saying something or using a clear verbal cue, such as a question, to signal to the child that it is their turn to speak.	The caregiver makes a comment on the play episode such as, “My car is going fast.” And then looks at the child to signal it is their turn to speak. The caregiver then waits at least 3 s to give the child time to respond.	The caregiver makes a comment on the play such as, “your car is going very fast” and immediately follows with another comment.
Verbal expansions	The caregiver adds on to what the child says to provide additional language exposure in the verbal interaction. The caregiver repeats what the child says before adding on.	The child says the word “boat.” The caregiver follows this by saying, “the boat is floating on the water.”	Caregiver acknowledges the child’s speech without adding on to the communication or simply repeats the word.
Modeling of target words through natural conversation	The caregiver uses the target word in a natural conversation to promote acquisition of the word.	“The car is driving to the house” “I wonder what the cake is going to taste like.”	Pairing of word use with strategies from prompting hierarchy such as use of a choice question.

**Table 4:** Naturalistic Book Reading Strategies.

Step or Strategy	Definition	Example
Introduces the book	The caregiver shows the title of the book and reads it aloud.	Today we will be reading *The Republic*
Provides feedback	Caregiver provides praise and/or acknowledges the child attending to the book.	I can tell you are really looking at that picture. I really like how you’re listening to the story.
Provides verbal expansions	When the child references the book in speech, the caregiver acknowledges the child and adds on to what they said.	Child says “ball” The caregiver replies by saying “the ball is rolling down the hill”
Use of EMT prompting strategies	Wait time Open-ended question Choice question Mand model	See Table 3
Modeling of target words	Caregiver points to a picture of the target word and uses the target word.	“The boat is in the water.”
Provides wait time between pages to give the child the opportunity to say something	After finishing a page, the caregiver waits three seconds before beginning the next page to give the child time to comment.	The caregiver finishes reading the page, then pauses three seconds before turning the page.
Praises/thanks child at the end of the reading task	Caregiver provides direct praise or thanks to the child immediately after the book ends.	Thank you for reading with me; I really enjoyed this. I could tell you were listening to the story the entire time. I had so much fun reading with you.
Redirects child when distracted	Focuses child’s attention by using an attention-getting strategy involving verbalization and gesture	Look at the bird? Followed by pointing to the board Take a look. Followed by pointing to the image.

**Table 5:** Descriptive Summaries of Caregiver Target Variables Across Phases.

Dyad	Reading Fidelity During Baseline	Play Fidelity During Baseline	Total Fidelity During Baseline	Reading Fidelity During Intervention	Play Fidelity During Intervention	Total Fidelity During Intervention	Reading Fidelity During Maintenance	Play Fidelity During Maintenance	Total Fidelity During Maintenance
Lucy & Gianna	*M* = 33%, range = 27–36%	*M* = 19%, range = 14–29%	*M* = 28%, range = 22–23%	*M* = 91%, range = 73–100%	*M* = 58%, range = 14–82%	*M* = 79%, range = 50–94%	*	*	*
Mary & Elias	*M* = 40%, range = 27–55%	*M* = 29% **	*M* = 27%, range = 17–33%	*M* = 77%, range = 64–100%	*M* = 55%, range = 29–57%	*M* = 68%, range = 61–83%	*	*	*
Theresa & Anne	*M* = 51%, range = 36–64%	*M* = 30%, range = 14–43%	*M* = 44%, range = 28–61%	*M* = 80%, range = 55–100%	*M* = 57%, range = 57–57%	*M* = 67%, range = 56–72%	*	*	*
Cheryl & Skyler	*M* = 71%, range = 45–91%	*M* = 25%, range = 14–57%	*M* = 53%, range = 33–72%	*M* = 97%, range = 73–100%	*M* = 73%, range = 43–86%	*M* = 88%, range = 72–94%	*M* = 98%, range = 91–100%	*M* = 71%, range = 57–86%	*M* = 88%, range = 78–94%

Note: * Indicates that the dyad did not enter the delayed maintenance phase. ** Indicates that as there was only one probe of play data for Mary and Elias in the baseline phase, a range could not be calculated.

**Table 6:** Descriptive Summaries of Child Target Variables Across Phases.

Dyad	Rate of Target Word Use During Reading in Baseline	Rate of Target Word Use During Play in Baseline	Total Rate of Target Word Use in Baseline	Rate of Target Word Use During Reading in Intervention	Rate of Target Word Use During Play in Intervention	Total Rate of Target Word Use in Intervention	Rate of Target Word Use During Reading in Maintenance	Rate of Target Word Use During Play in Maintenance	Total Rate of Target Word Use in Maintenance
Lucy & Gianna	*M* = 0.00, range = 0.00–0.00	*M* = 0.03, range = 0.00–0.08	*M* = 0.02, range = 0.00–0.05	*M* = 0.63, range = 0.22–1.00	*M* = 0.25, range = 0.00–0.59	*M* = 0.38, range = 0.14–0.59	*	*	*
Mary & Elias	*M* = 0.25, range = 0.10–0.43	*M* = 0.00 **	*M* = 0.24, range = 0.08–0.43	*M* = 0.75, range = 0.00–4.00	*M* = 0.62, range = 0.00–2.00	*M* = 0.26, range = 0.00–0.56	*	*	*
Theresa & Anne	*M* = 0.06, range = 0.00–0.33	*M* = 0.20, range = 0.00–0.50	*M* = 0.12, range = 0.00–0.30	*M* = 0.60, range = 0.30–1.00	*M* = 1.50, range = 1.00–2.00	*M* = 0.90, range = 0.73–1.18	*	*	*
Cheryl & Skyler	*M* = 0.29, range = 0.00–0.86	*M* = 0.27, range = 0.00–1.50	*M* = 0.27, range = 0.00–0.55	*M* = 0.89, range = 0.29–1.50	*M* = 0.74, range = 0.00–1.50	*M* = 0.79, range = 0.25–1.56	*M* = 1.27, range = 0.86–1.50	*M* = 1.31, range = 0.75–2.50	*M* = 1.29, range = 1.00–1.63

Note: * Indicates that the dyad did not enter the delayed maintenance phase. ** Indicates that as there was only one probe of play data for Mary and Elias in the baseline phase, a range could not be calculated.

**Table 7:** Interobserver Agreement Data for Target Variables.

Dyad	Fidelity in Reading	Fidelity in Play	Child Word Use During Reading	Child Word Use During Play	Coaching Fidelity
Lucy & Gianna	*M* = 87%, range, 73–100%	*M* = 86%, range, 50–100%	*M* = 100%, range, 100–100%	*M* = 85%, range, 40–100%	*M* = 100%, range, 100–100%
Mary & Elias	*M* = 87%, range, 43–100%	*M* = 97%, range, 80–100%	*M* = 88%, range, 50–100%	*M* = 96%, range, 75–100%	*M* = 97%, range, 88–100%
Theresa & Anne	*M* = 91%, range, 71–100%	*M* = 86%, range, 50–100%	*M* = 80%, range, 50–100%	*M* = 83%, range, 50–100%	*M* = 100%, range, 100–100%
Cheryl & Skyler	*M* = 82%, range, 40–100%	*M* = 84%, range, 20–100%	*M* = 86%, range, 60–100%	*M* = 96%, range, 60–100%	*M* = 97%, range, 100–100%

## Data Availability

All data generated and analyzed during this study are included in this published article.

## Supplementary Materials

The following supporting information can be downloaded at https://www.mdpi.com/article/10.3390/bs16081311/s1, Figure S1: Play Fidelity Scoring Form. Note: Adapted from Kim et al. (2020); Figure S2: Reading Fidelity Scoring Form. Note: Adapted from Akemoglu et al. (2022) and Dillehay (2023); Figure S3: Coaching Fidelity Scoring Form; Figure S4: Child Word Use Scoring Form; Figure S5: Caregiver Reading Checklist; Figure S6: Caregiver Play Checklist; Table S1: Intervention Targets; Table S2: SMD and Tau-U Effect Sizes for Caregiver Fidelity Variables; Table S3: Tau-U Effect Size Calculations for Child Word Use.

## References

[B1-behavsci-16-01311] Akemoglu Y., Hinton V., Laroue D., Jefferson V. (2022). A parent-implemented shared reading intervention via telepractice. Journal of Early Intervention.

[B2-behavsci-16-01311] Akemoglu Y., Meadan H., Towson J. (2020). Embedding naturalistic communication teaching strategies during shared interactive book reading for preschoolers with developmental delays: A guide for caregivers. Early Childhood Education Journal.

[B3-behavsci-16-01311] American Psychiatric Association (2013). Diagnostic and statistical manual of mental disorders.

[B4-behavsci-16-01311] Barnes E., Puccioni J. (2017). Shared book reading and preschool children’s academic achievement: Evidence from the early Childhood Longitudinal Study—Birth cohort. Infant and Child Development.

[B5-behavsci-16-01311] Bracken S. S., Fischel J. E. (2008). Family reading behavior and early literacy skills in preschool children from low-income backgrounds. Early Education and Development.

[B6-behavsci-16-01311] Clemens L. F., Kegel C. A. T. (2020). Unique contribution of shared book reading on adult-child language interaction. Journal of Child Language.

[B7-behavsci-16-01311] Dillehay K. M. (2023). Dosage, fidelity, and child outcomes in a small randomized controlled trial of EMT en Español. Doctoral dissertation.

[B8-behavsci-16-01311] Farrant B. M., Zubrick S. R. (2013). Parent–child book reading across early childhood and child vocabulary in the early school years: Findings from the Longitudinal Study of Australian Children. First Language.

[B9-behavsci-16-01311] Fenson L. (2002). Macarthur communicative development inventories: User’s guide and technical manual.

[B10-behavsci-16-01311] Ferguson J., Dounavi K., Craig E. (2022). The efficacy of using telehealth to coach parents of children with autism spectrum disorder on how to use naturalistic teaching to increase mands, tacts and intraverbals. Journal of Developmental and Physical Disabilities.

[B11-behavsci-16-01311] García-Grau P., Martínez-Rico G., Cañadas M., González-García R. J. (2024). Social validity of telepractice in families with children with autism. Research in Autism Spectrum Disorders.

[B12-behavsci-16-01311] Garnett R., Davidson B., Eadie P. (2022). Telepractice delivery of an autism communication intervention program to parent groups. Research in Autism Spectrum Disorders.

[B13-behavsci-16-01311] Garnett R., Davidson B., Eadie P., Clarke K., Aggarwal D. (2019). Telepractice delivery of an autism communication intervention to parent groups: A feasibility study. Journal of Clinical Practice in Speech-Language Pathology.

[B14-behavsci-16-01311] Gilkerson J., Richards J. A., Topping K. J. (2017). The impact of book reading in the early years on parent–child language interaction. Journal of Early Childhood Literacy.

[B15-behavsci-16-01311] González-García R. J., Martínez-Rico G., Escorcia-Mora C., García-Grau P. (2023). A Bibliometric study on the social validity of telepractice in autism spectrum disorder. International Journal of Environmental Research and Public Health.

[B16-behavsci-16-01311] Hancock T. B., Kaiser A. P. (2002). The effects of trainer-implemented enhanced milieu teaching on the social communication of children with autism. Topics in Early Childhood Special Education.

[B17-behavsci-16-01311] Hedges L. V., Pustejovsky J. E., Shadish W. R. (2012). A standardized mean difference effect size for single case designs. Research Synthesis Methods.

[B18-behavsci-16-01311] Hemmeter M. L., Kaiser A. (1994). Enhanced milieu teaching: Effects of parent-implemented language intervention. Journal of Early Intervention.

[B19-behavsci-16-01311] Holmes E., Willoughby T. (2005). Play behaviour of children with autism spectrum disorders. Journal of Intellectual & Developmental Disability.

[B20-behavsci-16-01311] Hume K., Steinbrenner J. R., Odom S. L., Morin K. L., Nowell S. W., Tomaszewski B., Szendrey S., McIntyre N. S., Yücesoy-Özkan S., Savage M. N. (2021). Evidence-based practices for children, youth, and young adults with autism: Third generation review. Journal of Autism and Developmental Disorders.

[B21-behavsci-16-01311] Kaderavek J. (2002). Shared storybook reading as an intervention context. American Journal of Speech-Language Pathology.

[B22-behavsci-16-01311] Kaiser A. P., Hancock T. B., Nietfeld J. P. (2000). The effects of parent-implemented enhanced milieu teaching on the social communication of children who have autism. Early Education and Development.

[B23-behavsci-16-01311] Kaiser A. P., Hester P. P. (1994). Generalized effects of enhanced milieu teaching. Journal of Speech, Language, and Hearing Research.

[B24-behavsci-16-01311] Kim S., Kang V. Y., McLeod R. H. (2020). Effects of enhanced milieu teaching with book reading for children with autism spectrum disorder. Education and Training in Autism and Developmental Disabilities.

[B25-behavsci-16-01311] Kratochwill T. R., Hitchcock J., Horner R. H., Levin J. R., Odom S. L., Rindskopf D. M., Shadish W. R. (2010). Single-case designs technical documentation. *What Works Clearinghouse*.

[B26-behavsci-16-01311] Ledford J. R., Gast D. L. (2018). Single case research methodology.

[B27-behavsci-16-01311] Lovaas O. I. (1987). Behavioral treatment and normal educational and intellectual functioning in young autistic children. Journal of Consulting and Clinical Psychology.

[B28-behavsci-16-01311] Marchman V. A., Fernald A. (2008). Speed of word recognition and vocabulary knowledge in infancy predict cognitive and language outcomes in later childhood. Developmental Science.

[B29-behavsci-16-01311] Mathers S. J., Hodgkiss A., Kolancali P., Booton S., Wang Z., Murphy V. A. (2024). Comparing parent-child interaction during wordless book reading, print book reading and imaginative play. Journal of Child Language.

[B30-behavsci-16-01311] McLeod R. H., Hardy J. K., Kaiser A. P. (2017). The effects of play-based intervention on vocabulary acquisition by preschoolers at risk for reading and language delays. Journal of Early Intervention.

[B31-behavsci-16-01311] Meadan H., Chung M. Y., Sands M. M., Snodgrass M. R. (2020). The cascading coaching model for supporting service providers, caregivers, and children. The Journal of Special Education.

[B32-behavsci-16-01311] Meadan H., Snodgrass M. R., Meyer L. E., Fisher K. W., Chung M. Y., Halle J. W. (2016). Internet-based parent-implemented intervention for young children with autism: A pilot study. Journal of Early Intervention.

[B33-behavsci-16-01311] Movahedazarhouligh S. (2021). Parent-implemented interventions and family-centered service delivery approaches in early intervention and early childhood special education. Early Child Development and Care.

[B34-behavsci-16-01311] Noble C., Sala G., Peter M., Lingwood J., Rowland C., Gobet F., Pine J. (2019). The impact of shared book reading on children’s language skills: A meta-analysis. Educational Research Review.

[B35-behavsci-16-01311] Oono I. P., Honey E. J., McConachie H. (2013). Parent-mediated early intervention for young children with autism spectrum disorders (ASD). The Cochrane Database of Systematic Reviews.

[B36-behavsci-16-01311] Pacia C., Holloway J., Gunning C., Lee H. (2022). A systematic review of family-mediated social communication interventions for young children with autism. Review Journal of Autism and Developmental Disorders.

[B37-behavsci-16-01311] Pacione L. (2022). Telehealth-delivered caregiver training for autism: Recent innovations. Frontiers in Psychiatry.

[B38-behavsci-16-01311] Parker R. I., Vannest K. J., Davis J. L., Sauber S. B. (2011). Combining nonoverlap and trend for single-case research: Tau-U. Behavior Therapy.

[B39-behavsci-16-01311] Piaget J. (1952). Play, dreams and imitation in childhood.

[B40-behavsci-16-01311] Poole M. E., Fettig A., McKee R. A., Gauvreau A. N. (2022). Inside the virtual visit: Using tele-intervention to support families in early intervention. Young Exceptional Children.

[B41-behavsci-16-01311] Prelock P. J., Nelson N. W. (2012). Language and communication in autism: An integrated view. Pediatric Clinics.

[B42-behavsci-16-01311] Pustejovsky J. E., Chen M., Grekov P., Swan D. M. (2024). Single-case effect size calculator *(Version 0.7.3) [Web application]*.

[B43-behavsci-16-01311] Reichow B., Volkmar F. R., Cicchetti D. V. (2008). Development of the evaluative method for evaluating and determining evidence-based practices in autism. Journal of Autism and Developmental Disorders.

[B44-behavsci-16-01311] Rickards A. L., Walstab J., Wright-Rossi R. A., Simpson J., Reddihough D. (2009). One-year follow-up of the outcome of a randomized controlled trial of a home-based intervention programme for children with autism and developmental delay and their families. Child: Care, Health and Development.

[B45-behavsci-16-01311] Roulstone S., Law J., Rush R., Clegg J., Peters T. (2011). Investigating the role of language in children’s early educational outcomes.

[B46-behavsci-16-01311] Rutter M. (2003). Social communication questionnaire.

[B47-behavsci-16-01311] Schaefer J. M., Andzik N. R. (2021). Evaluating behavioral skills training as an evidence-based practice when training parents to intervene with their children. Behavior Modification.

[B48-behavsci-16-01311] Schreibman L., Dawson G., Stahmer A. C., Landa R., Rogers S. J., McGee G. G., Kasari C., Ingersoll B., Kaiser A. P., Bruinsma Y., McNerney E., Wetherby A., Halladay A. (2015). Naturalistic developmental behavioral interventions: Empirically validated treatments for autism spectrum disorder. Journal of Autism and Developmental Disorders.

[B49-behavsci-16-01311] Sénéchal M., LeFevre J.-A. (2014). Continuity and change in the home literacy environment as predictors of growth in vocabulary and reading. Child Development.

[B50-behavsci-16-01311] Sénéchal M., Pagan S., Lever R., Ouellette G. P. (2008). Relations among the frequency of shared reading and 4-year-old children’s vocabulary, morphological and syntax comprehension, and narrative skills. Early Education and Development.

[B51-behavsci-16-01311] Slocum T., Joslyn P., Nichols B., Pinkelman S. (2022a). Revisiting an analysis of threats to internal validity in multiple baseline designs. Perspectives on Behavior Science.

[B52-behavsci-16-01311] Slocum T., Pinkelman S., Joslyn P., Nichols B. (2022b). Threats to internal validity in multiple-baseline design variations. Perspectives on Behavior Science.

[B53-behavsci-16-01311] Towson J. A., Akemoglu Y., Watkins L., Zeng S. (2021). Shared interactive book reading interventions for young children with disabilities: A systematic review. American Journal of Speech-Language Pathology.

[B54-behavsci-16-01311] Trivette C. M., Dunst C. J., Hamby D. W. (2010). Influences of family-systems intervention practices on parent-child interactions and child development. Topics in Early Childhood Special Education.

[B55-behavsci-16-01311] van Noorden L., Gardiner S., Waddington H. (2026). Parent-mediated naturalistic developmental behavioral interventions for young autistic children: A systematic literature review of single-case research. Review Journal of Autism and Developmental Disorders.

[B56-behavsci-16-01311] Vivanti G., Zhong H. N., Vivanti G., Bottema-Beutel K., Turner-Brown L. (2020). Naturalistic developmental behavioral interventions for children with autism. Clinical guide to early interventions for children with autism.

[B57-behavsci-16-01311] Vygotsky L. S. (2016). Play and its role in the mental development of the child (N. Veresov, & M. Barrs, Trans.). International Research in Early Childhood Education.

[B58-behavsci-16-01311] Ward-Horner J., Sturmey P. (2012). Component analysis of behavior skills training in functional analysis. Behavioral Interventions.

[B59-behavsci-16-01311] Watson P. J., Workman E. A. (1981). The non-concurrent multiple baseline across-individuals design: An extension of the traditional multiple baseline design. Journal of Behavior Therapy and Experimental Psychiatry.

