# Supplementary material for: Caregiver-Mediated Enhanced Milieu Teaching with Shared Book Reading Using Telepractice-Based Coaching
Source: Behav Sci (Basel). 2026 Aug 2;16(8):1311. doi: 10.3390/bs16081311 (PMC13510035; doi:10.3390/bs16081311)
Supplement: Supplementary file 1 [file behavsci-16-01311-s001.zip › behavsci-4402752-File S1.pdf]

behavioral sciences

MDPI

File S1. Additional Tables

Table S1. Intervention Targets.

Child	Primary Target Theme	Primary Target Words	Generalization Target Theme	Generalization Target Words
Gianna	Ocean	Seal, shark, eel	Princess	Fairy, coach, slipper
Elias	Zoo	Elephant, lion, polar bear	Aquarium	Jellyfish, penguin, otter
Anne	Animals (ostriches)	Ostrich, baboon, insect, jackal	Animals (ocean)	Shell, starfish, urchin, eel
Skyler	Vehicles (backhoe loader)	Dipper, bucket, tires, dumpster, stump	Vehicles (garbage truck)	Trash, hopper, crush, smash, garbage

Table S2. SMD and Tau-U Effect Sizes for Caregiver Fidelity Variables.

Participant	Variable	Effect Size	Standard Error	Confidence Interval	Baseline Standard Deviation	TAU-U
Lucy	Reading fidelity	6.63	1.96	95% CI: [2.78, 10.48]	5.20	0.92
Lucy	Play fidelity	2.80	1.02	95% CI: [0.80, 4.79]	8.66	0.83
Lucy	Total fidelity	5.56	1.72	95% CI: [2.19, 8.93]	5.51	0.96
Mary	Reading fidelity	2.89	0.88	95% CI: [1.17, 4.60]	11.51	1.09
Mary	Play fidelity	*	*	*	*	*
Mary	Total fidelity	12.30	6.88	95% CI: [−1.19, 25.80]	5.85	1.08
Theresa	Reading fidelity	1.83	0.62	95% CI: [0.62, 3.05]	16.70	0.80
Theresa	Play fidelity	0.94	0.54	95% CI: [−0.12, 2.00]	16.77	0.39
Theresa	Total fidelity	2.58	0.72	95% CI: [1.17, 3.99]	9.32	0.77
Cheryl	Reading fidelity	1.58	0.43	95% CI: [0.73, 2.42]	19.31	0.88
Cheryl	Play fidelity	2.26	0.58	95% CI: [1.13, 3.40]	17.91	0.94
Cheryl	Total fidelity	2.36	0.57	95% CI: [1.25, 3.46]	14.39	0.92

Note: * Indicates that there were insufficient data to calculate an effect size. Tau-U may exceed 1.0 due to baseline correction.

Table S3. Tau-U Effect Size Calculations for Child Word Use.

Participant	Variable	TAU-U
Gianna	Word use during reading	1.00
Gianna	Word use during play	0.58
Gianna	Word use total	0.96
Elias	Word use during reading	0.21
Elias	Word use during play	*
Elias	Word use total	0.21
Anne	Word use during reading	0.85
Anne	Word use during play	0.62
Anne	Word use total	0.68
Skyler	Word use during reading	0.73
Skyler	Word use during play	0.27
Skyler	Word use total	0.72

Note: * Indicates that there were insufficient data to calculate an effect size. Tau-U may exceed 1.0 due to baseline correction.
