# Supplementary material for: Caregiver-Mediated Enhanced Milieu Teaching with Shared Book Reading Using Telepractice-Based Coaching
Source: Behav Sci (Basel). 2026 Aug 2;16(8):1311. doi: 10.3390/bs16081311 (PMC13510035; doi:10.3390/bs16081311)
Supplement: Supplementary file 1 [file behavsci-16-01311-s001.zip › behavsci-4402752-File S2.pdf]

behavioral sciences

MDPI

File S2. Fidelity Scoring Forms

Baseline and Follow-up criteria	Intervention Criteria
Correct toys provided and set up in proper area	Correct toy set and book used in play area
No use or modeling of target words	Use of all target words in a conversation at least twice in a session
No prompts or reinforcements of target words	Use of a system of least to most prompts to deliver the EMT strategies for target words at least one per session
	Use of EMT strategies at child's language level
	Provides verbal expansion for each target word at least once
	EMT strategeis used when child is attending
	Immediate access to target materials when child uses target word

Figure S1. Play Fidelity Scoring Form. Note: Adapted from Kim et al. (2020).

Book Reading Fidelity Checklist			Total points
Sits in a distraction free area		1p	
Introduces book by title		1p	
Models target word at least once		1p	
Use of all EMT prompting strategeis at least once per session			
Time-delay		1p	
Open-ended Question		1p	
Choice Question		1p	
Mand-Model		1p	
Provide feedback/encouragement at least 2 times during session 2p			
Provide correction when necessary 1p*			
Redirects child when child is distracted 1p*			
Provides verbal expansion at least twice in a session 2p			
Praises or thanks child at the end of the reading activity 1p			
Must recieve at least 11 points to reach criteria			
* indicates that these are optional and may not occur in every session			

Figure S2. Reading Fidelity Scoring Form. Note: Adapted from Akemoglu et al. (2022) and Dillehay (2023).

Coaching Procedural Fidelity			
	Yes	No	Total of 8
Greet Parent			
State the date of the video you are reviewing			
Ask parent what they think went well, and what they think they can improve on.			
Show parent what they did correctly			
Show parent an area of improvment			
Model what can be done instead to improve			
Make a plan with parent for next session			
Ask parent if they have any questions			

Figure S3. Coaching Fidelity Scoring Form.

EMT Intervention Data Recording Form							
Dyad (pseudonyms) :							
Coder name:							
Date video was uploaded:							
Date video was coded:							
Instructions: Write the target word ins the corresponding box you are tracking. Make a tally mark for each unprompted occurance of the word. Unprompted word use occurs when the child says the target word either a) spontaneously, b) within 3 seconds of parent natural conversation use or model, c) within 3 seconds of parent initating a wait time, d) within 3 seconds of parent using an open ended question. Do NOT count child word use that occurs after a mand model or choice question.							
See accompanying definitions sheet for strategy definitions and examples.							
Example of target response: If target word is "boat"- child says "boat" within three seconds of parent use, a wait time, or an open-ended question.							
Non-example: if target word is "boat"- child says "bo" (approximation),or child says "boat" following parent implentation of a choice question or mand-model.							
Target word 1:				Time stamps:			
Target word 2:				Time stamps:			
Target word 3:				Time stamps:			
Target word 4:				Time stamps:			
Target word 5:				Time stamps:			

Figure S4. Child Word Use Scoring Form.
