# Supplementary material for: Caregiver-Mediated Enhanced Milieu Teaching with Shared Book Reading Using Telepractice-Based Coaching
Source: Behav Sci (Basel). 2026 Aug 2;16(8):1311. doi: 10.3390/bs16081311 (PMC13510035; doi:10.3390/bs16081311)
Supplement: Supplementary file 1 [file behavsci-16-01311-s001.zip › behavsci-4402752-File S3.pdf]

behavioral sciences

MDPI

File S3. Social Validity Survey

Post-Study Survey		
Directions: The following questions are about your experience participating in this study. Please answer them as honestly as you can.		
1. Did you find the intervention helpful for your child?	Yes	No
2. Did you find the training adequate?	Yes	No
3. Do you think your child learned during this study?	Yes	No
4. Was the study worth it?	Yes	No
5. Are you satisfied with the study and the results?	Yes	No
What about the study did you find helpful for your child's learning? What did you like?		
What about the study did you find unhelpful or stressful? What did you not like?		
