# Supplementary material for: Caregiver-Mediated Enhanced Milieu Teaching with Shared Book Reading Using Telepractice-Based Coaching
Source: Behav Sci (Basel). 2026 Aug 2;16(8):1311. doi: 10.3390/bs16081311 (PMC13510035; doi:10.3390/bs16081311)
Supplement: Supplementary file 1 [file behavsci-16-01311-s001.zip › behavsci-4402752-File S4.pdf]

behavioral sciences

MDPI

File S4. Caregiver Checklists

Book Reading Checklist

1. Pick a quiet and distraction free area ☐
2. Introduce the book. Say the title and author. ☐
3. Model each target word at least 1 time. ☐
4. Use wait time at least 1 time. ☐
5. Use an open-ended question at least 1 time. ☐
6. Use a choice question at least 1 time. ☐
7. Use a say prompt at least 1 time. ☐
8. Provide feedback or encouragement at least 2 times. ☐
9. Expand on what your child says at least 2 times. ☐
10. Thank your child for reading with you. ☐

Only provide correction or redirection if necessary.

Figure S5. Caregiver Reading Checklist.

Play Time Checklist

1. Correct toy set in the play area. ☐
2. Use all target vocabulary words in conversation or by modeling at least twice. ☐
3. Use EMT prompts in the correct order (wait time -> open-ended question -> choice question -> say prompt). ☐
4. Use child-friendly language. ☐
5. Expand on each target word at least once. ☐
6. Use the strategies when your child is paying attention. ☐
7. Give your child access to the toy when they request it correctly. ☐

Figure S6. Caregiver Play Checklist.
